# Histone acetyltransferases: challenges in targeting bi-substrate enzymes

**DOI:** 10.1186/s13148-016-0225-2

**Published:** 2016-05-26

**Authors:** Hannah Wapenaar, Frank J. Dekker

**Affiliations:** Department of Pharmaceutical Gene Modulation, University of Groningen, Antonius Deusinglaan 1, 9713 AV Groningen, The Netherlands

**Keywords:** Histone acetyltransferases, Epigenetics, Lysine acetylation, HAT inhibitors, Catalytic mechanism, Inhibitor kinetics

## Abstract

Histone acetyltransferases (HATs) are epigenetic enzymes that install acetyl groups onto lysine residues of cellular proteins such as histones, transcription factors, nuclear receptors, and enzymes. HATs have been shown to play a role in diseases ranging from cancer and inflammatory diseases to neurological disorders, both through acetylations of histone proteins and non-histone proteins. Several HAT inhibitors, like bi-substrate inhibitors, natural product derivatives, small molecules, and protein–protein interaction inhibitors, have been developed. Despite their potential, a large gap remains between the biological activity of inhibitors in in vitro studies and their potential use as therapeutic agents. To bridge this gap, new potent HAT inhibitors with improved properties need to be developed. However, several challenges have been encountered in the investigation of HATs and HAT inhibitors that hinder the development of new HAT inhibitors. HATs have been shown to function in complexes consisting of many proteins. These complexes play a role in the activity and target specificity of HATs, which limits the translation of in vitro to in vivo experiments. The current HAT inhibitors suffer from undesired properties like anti-oxidant activity, reactivity, instability, low potency, or lack of selectivity between HAT subtypes and other enzymes. A characteristic feature of HATs is that they are bi-substrate enzymes that catalyze reactions between two substrates: the cofactor acetyl coenzyme A (Ac-CoA) and a lysine-containing substrate. This has important—but frequently overlooked—consequences for the determination of the inhibitory potency of small molecule HAT inhibitors and the reproducibility of enzyme inhibition experiments. We envision that a careful characterization of molecular aspects of HATs and HAT inhibitors, such as the HAT catalytic mechanism and the enzyme kinetics of small molecule HAT inhibitors, will greatly improve the development of potent and selective HAT inhibitors and provide validated starting points for further development towards therapeutic agents.

## Background

**Fig. 1:**
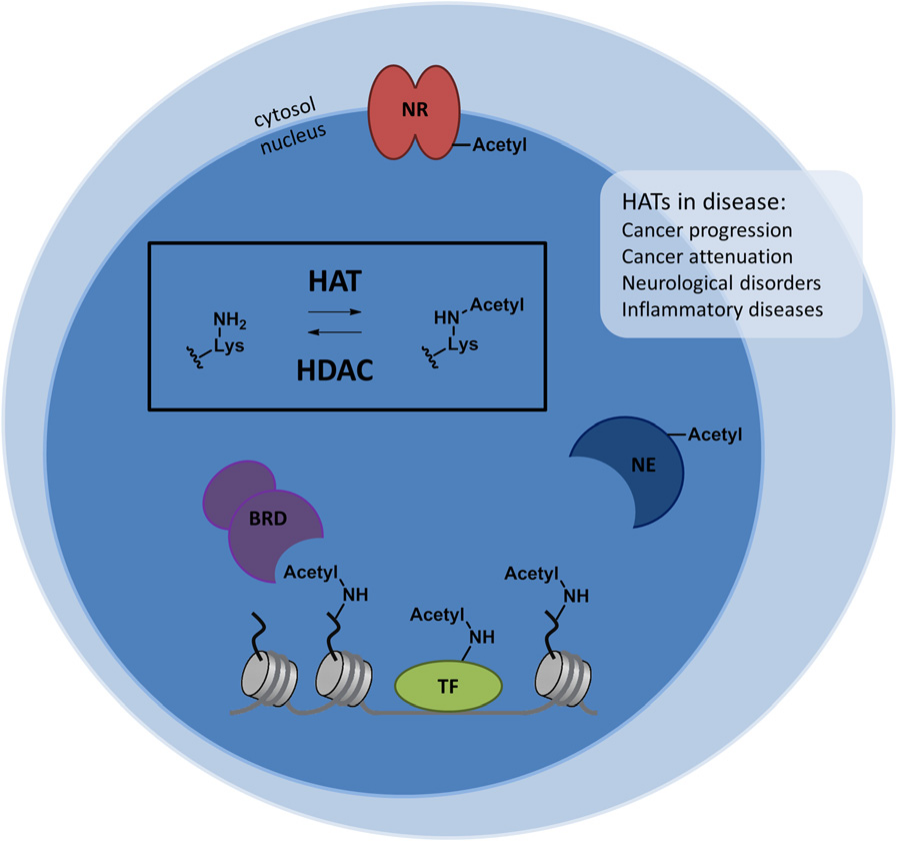
Lysine acetylation is balanced by histone acetyltransferases (HATs) and histone deacetylases (HDACs). Acetylation of lysine residues on the histone tails that protrude from the histone–DNA complex modifies the chromatin structure of the DNA, which allows transcription factors to bind. The transcription factors themselves can be acetylated, which influences promotor activity and specificity. Lysine acetylation of enzymes or nuclear receptors can influence their function. Bromodomain-containing proteins will bind to the acetylated lysine residues. Through lysine acetylations, HATs are involved in many different diseases such as cancer, inflammatory diseases, and neurological disorders. *NR* nuclear receptor, *BRD* bromodomain, *NE* nuclear enzyme, *TF* transcription factor

Many diseases are connected to aberrant patterns of post-translational modifications of cellular proteins such as acetylations of lysine residues [1, 2]. Several cellular proteins including histones, transcription factors, nuclear receptors, and enzymes are subject to lysine acetylations, which play a key role in the regulation of their functions [3]. Acetylations of lysine residues on histones are involved in epigenetic regulation of gene transcription [4, 5]. Apart from histones, lysine acetylations of transcription factors, such as Myc proto-oncogene protein (c-MYC), p53, and nuclear factor kappa-light-chain-enhancer of activated B cells (NF-κB), have been shown to influence their promotor activities and specificities [6–8]. Lysine acetylations of enzymes or nuclear receptors play important regulatory roles in their function [9, 10]. Furthermore, lysine acetylations are involved in protein–protein interactions via bromodomains [5] (Fig. 1). Reversible lysine acetylations are mediated by histone acetyltransferases (HATs), which install acetyl groups onto lysine residues, and histone deacetylases (HDACs), which remove acetyl groups from lysine residues (Fig. 1). HDACs have been studied extensively, mainly for their role in cancer, and two HDAC inhibitors are currently on the market [9, 10]. In contrast, no clinical applications of HATs have been described until now. Nevertheless, HATs have been shown to play a role in diseases ranging from cancer and inflammatory diseases to neurological disorders [11–13].

In cancer, HATs have been shown to suppress as well as to stimulate tumor growth and disease progression. Acetylation of histones can lead to a less condensed DNA and therefore more gene transcription [5]. If these genes are (proto-)oncogenes, hyperacetylation might aid in cancer progression, whereas less acetylation might protect against disease. Indeed, histone hyperacetylation was found in hepatocellular carcinoma, and acetylation of a specific lysine on histone H3 (H3K18) was correlated with prostate cancer recurrence [14, 15]. Lower levels of H3K18 were shown to be advantageous for glioma patients [16]. However, when investigating the HATs themselves, they were found to have opposite effects, even within the same type of cancer. For example, the KAT3B HAT gene was suggested to function as tumor suppressor gene in colorectal cancer [17], but high levels of KAT3B mRNA were correlated with progression of the disease [18]. Also in non-histone acetylation, HATs seem to exert counteracting effects. The HATs KAT2A, 2B, and 5 acetylate the oncogene c-MYC leading to increased stability of the c-MYC protein, which may lead to cancer progression [6]. In contrast, KAT2B also acetylates the tumor suppressor protein p53 and activates its transcriptional activity, suggesting a protective function for KAT2B [19]. The exact role of HATs in cancer and the regulatory factors influencing HATs are therefore still under investigation.

Histone acetylation and HAT activity are involved in inflammatory diseases. The HATs KAT3A and KAT3B were shown to activate the expression of pro-inflammatory interleukins like IL-5, IL-8, and IL-4 [20–22]. HATs also function as cofactors of NF-κB and activate its transcriptional activity [23, 24]. NF-κB itself is acetylated by HATs on various positions, which influences promotor activity and specificity [8]. In diabetic type-2 patients, inflammatory processes can increase insulin resistance. NF-κB was shown to be recruited to gene promotors under diabetic conditions, and an increase of histone acetylation was observed in monocytes of diabetic patients [25]. An increase in the activity of HATs was observed in blood monocytes of patients with asthma [26]. In pulmonary fibrosis, it was shown that inhibiting the KAT3A/β-catenin interaction attenuated and even reversed disease by influencing the Wnt signaling pathway [27]. HATs have been shown to activate inflammatory signaling and may therefore be promising targets for treatment of inflammatory diseases. On the other hand, however, a study on KAT2B showed that this HAT was essential for inflammation-induced post-ischemic arteriogenesis, suggesting that activation of KAT2B can aid in recovery after ischemic events such as stroke or myocardial infarction [28].

Genetic mutations or deletions of HAT genes have severe consequences for neuronal development and function [13]. A mutation in the KAT3A and KAT3B genes causes the Rubinstein–Taybi syndrome. This disease is characterized by growth impairment, mental retardation, and typical morphologies like broad thumbs and halluces and distinct facial features [29]. Therefore, it is suggested that HATs play a role in the maturation of neurons in embryonic development, memory, learning, and even skeleton formation.

Most research on HATs and their role in diseases depends on genetically modified mice and cellular studies. These methods, however, have limitations. Knock-out mice, for example, need to be viable to be studied, and knock-out of many HAT genes is incompatible with life [30–32]. Immortalized cell lines may behave very differently from the diseased or healthy situation, and little information on the molecular level can be derived from these models. Therefore, drug discovery projects have been initiated to identify small molecule inhibitors of HAT activity that can be used for the development of research tools to study their functions as well as the exploration of their potential as targets for therapeutic interventions [33, 34]. Despite their potential, the development of small molecule inhibitors for HATs proved to be challenging and a large gap remains between the biological activity of inhibitors in in vitro studies and their use as therapeutic agents. To bridge this gap, new potent HAT inhibitors with improved properties need to be developed. However, several challenges have been encountered in the investigation of HATs and HAT inhibitors that hinder the development of new HAT inhibitors. In this review, we will discuss these challenges and we propose that careful investigation of the molecular aspects of HAT function and inhibition will give a solid starting point for the development of new potent and selective HAT inhibitors with therapeutic potential.

### The HAT enzymes—challenges in substrate specificity

The human HATs are classified as lysine (K) acetyltransferases (KATs). It should be noted that alternative nomenclature, as indicated in Table 1, is frequently used as well. Type B HATs (KAT1, HAT4) are cytoplasmic enzymes—they modify free histones in the cytoplasm just after their synthesis, upon which they are transported to the nucleus and integrated in newly synthesized DNA [35]. Type A HATs are (mainly) nuclear enzymes. They are responsible for acetylations of histones and non-histone proteins in the nucleus. Based on their sequence homology, most nuclear HATs can be assigned to families. The GNAT (Gcn5-related N-acetyltransferases) family consists of KAT2A and KAT2B. The MYST family (after the members MOZ, YBF2/SAS3, SAS2, and TIP60) is the largest family and consists of KAT5, 6A and 6B, 7, and 8. The p300/CBP family consists of KAT3A and 3B. Other HATs are the transcriptional co-activators, such as KAT4 and KAT12, and steroid receptor co-activators, such as KAT13A-D, that possess acetyltransferase activity next to their other functions.

**Table 1:** Histone acetyltransferases: families, subtypes, and alternative nomenclature frequently used

Family	Subtype	Other names frequently used
Cytoplasmic	KAT1	HAT1
	HAT4	NAA60
GNAT	KAT2A	Gcn5
	KAT2B	PCAF
MYST	KAT5	TIP60
	KAT6A	MOZ, MYST3
	KAT6B	MORF, MYST4
	KAT7	HBO1, MYST2
	KAT8	MOF, MYST1
p300/CBP	KAT3B	p300
	KAT3A	CBP
Transcription co-activators	KAT4	TAF1, TBP
	KAT12	TIFIIIC90
Steroid receptor co-activators	KAT13A	SRC1
	KAT13B	SCR3, AIB1, ACTR
	KAT13C	p600
	KAT13D	CLOCK

The HAT isoenzymes have various substrate specificities for histone or non-histone proteins. For example, the HATs KAT3A and 3B acetylate all four histone subtypes (histone H2A, H2B, H3, and H4), but KAT6A acetylates only histone H3 [36, 37] and KAT8 acetylates specifically lysine 16 on histone H4 (H4K16) [38]. This substrate specificity is modulated by the incorporation of HATs in large multi-subunit protein complexes [39]. For example, KAT8 operates through two evolutionary conserved protein complexes, the MSL-1 complex, and the MSL1v1 complex. The acetylation activity of these two protein complexes on histone H4 is identical, but acetylation of the non-histone target p53 differs dramatically [40]. It was also shown that recombinant KAT8, free of interactions with proteins from either complex, acetylated H2A and H3 as well as H4, in contrast to the specificity of the KAT8 protein complexes for H4K16 [41]. Also in the case of KAT2A, incorporation into its SAGA and Ada complexes influences the specificity and the catalytic activity towards its histone targets as well as its non-histone targets [42]. The influence of the HAT protein complexes on acetyltransferase activity and substrate specificity is one of the challenges that need to be addressed in the development of small molecule HAT inhibitors, considering that the activities of recombinant HAT enzymes may not reflect their in vivo activity. This may limit the translation from in vitro assays to in vivo disease models.

### HAT inhibitors—challenges in molecular properties

Parallel to functional studies on HATs, research has aimed at developing small molecule inhibitors as research tools or as potential therapeutic agents. Different approaches such as construction of HAT substrate mimics, research on natural products, and high throughput and virtual screening have been used to identify HAT inhibitors.

**Fig. 2:**
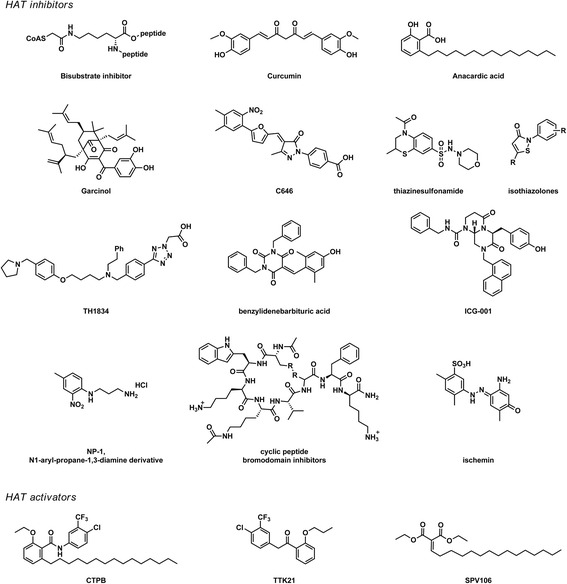
The current HAT inhibitors and activators. Bi-substrate inhibitors mimic the two HAT substrates: Ac-CoA, connected via a linker to a peptide resembling the lysine substrate. Garcinol, curcumin, and anacardic acid are natural product HAT inhibitors. Small molecule inhibitors C646 and thiazinesulfonamide were discovered from a virtual screening. A high throughput screening yielded isothiazolone derivatives. A pentamidine derivative, TH1834, and a benzylidene barbituric acid derivative were developed using a structure-based design. ICG-001 is a protein–protein interaction inhibitor and inhibits the interaction between KAT3A and β-catenin. HAT bromodomain inhibitors have been developed for KAT3A and KAT2B, including the natural product ischemin, a set of cyclic peptides and small molecule N1-aryl-propane-1,3-diamine derivatives. CTPB, TTK21, and SPV106 are salicylic acid-derived HAT activators. CTBP activates KAT3B, TTK21 activates both KAT3B and KAT3A, and SPV106 interestingly is a KAT2B activator and KAT3A/3B inhibitor

One class of inhibitors is the bi-substrate inhibitors. These inhibitors mimic the two HAT substrates: the cofactor acetyl coenzyme A (Ac-CoA) and a peptide resembling the lysine substrate, connected via a linker (Fig. 2). Bi-substrate inhibitors have been made for KAT2B, KAT3B, KAT5, and the yeast KAT5 homologue ESA1 [43, 44] and are very selective. They have been used as dead-end inhibitors that mimic the natural substrate but cannot be converted by the enzyme in kinetic studies [45]. However, due to their peptidic nature and their size, bi-substrate inhibitors suffer from poor metabolic stability and a lack of cell permeability, which limits their applications in cellular systems.

Several small molecule HAT inhibitors have been derived from natural products. Among others, garcinol, curcumin, and anacardic acid (Fig. 2) have shown to be HAT inhibitors [46–48]. However, these natural products and close derivatives are not selective between HATs and often have many other targets [49, 50]. Many natural product HAT inhibitors contain phenolic structures, which are prone to oxidation. As a result, it is often hard to determine whether the observed effects in advanced disease models are due to inhibition of HAT activity or due to anti-oxidant properties. For other natural products such as anacardic acid, their lipophilic and amphiphilic character is a limiting factor for further exploration and optimization. Nevertheless, promising cellular effects have been observed for this type of HAT inhibitors. The natural product HAT inhibitors garcinol and anacardic acid have been shown to sensitize cancer cells to irradiation [51, 52]. Garcinol suppressed proliferation of breast cancer cells and inhibited colon carcinogenesis in mice [53, 54]. Curcumin is indeed a HAT inhibitor, but its biological effect cannot be solely appointed to HAT inhibition. Curcumin is an anti-oxidant and additionally contains a Michael acceptor that can react with nucleophiles present in cells, like thiols or anions of alcohols [55]. It can, therefore, influence many processes in the cell, unrelated to HAT inhibitory activity. Nevertheless, curcumin is currently in clinical trials for many applications as a therapeutic agent, combination therapy, or dietary supplement (Table 2), although it must be noted that measurements on HATs or their activities are not included in the outcome parameters of these trials. Recently, promising results have been obtained with a structure-based design to improve natural product HAT inhibitors. Modification of the structure of pentamidine lead to the inhibitor TH1834, and a benzylidene barbituric acid derivative with improved selectivity and cell permeability was developed from garcinol [56, 57]. Thus, although natural products may suffer from undesired properties, they form excellent starting points for further development of HAT inhibitors (Fig. 2).

**Table 2:** HAT inhibitors and activators, their target histone acetyltransferases, and proposed target pathologies

	HATs	Proposed target pathologies	References
HAT inhibitors			
Bi-substrate inhibitors	Various	–	[43–45]
Garcinol	KAT3B	Breast cancer, colon carcinoma	[48, 52–54]
Curcumin	KAT3B	Cancer, inflammation, neurological disorders, cardiovascular disease, metabolic diseases^a^	[47], clinicaltrials.gov
Anacardic acid	Non-selective	Sensitizing cancer cells to irradiation	[46, 51]
TH1834	KAT5	Breast cancer	[56]
Benzylidene barbituric acid	KAT3B	Cell cycle arrest, increase in hypodiploid nuclei	[57]
Isothiazolones	various	Inhibition of cancer cell proliferation	[58–61]
Thiazinesulfonamide	KAT3B	–	[62]
C646	KAT3B	Prostate cancer, melanoma, leukemia, peritoneal fibrosis	[63–68]
ICG-001	KAT3A/β-catenin	Investigation of the KAT3A/β-catenin interaction in survivin gene transcription, colon carcinoma	[70, 71]
Ischemin (bromodomain inhibitor)	KAT2A and 2B, KAT3A and 3B	Myocardial ischemia	[73]
Cyclic peptide bromodomain inhibitors		Inhibitors of the tumor suppressor protein p53	[74]
N1-aryl-propane-1,3-diamine derivatives (bromodomain inhibitors)		HIV-1	[75]
HAT activators			
CTPB	KAT3B	–	[46]
TTK21	KAT3A and 3B	Neurogenesis and long-term memory, brain disease	[76]
Pentadecylidenemalonate	KAT2B	Conditioned fear, wound repair, cardiovascular disease, diabetes	[77]

^a^Curcumin is currently in clinical trials for many applications as a therapeutic agent, combination therapy, or dietary supplement, although measurements on HATs or their activities were not included in the outcome parameters of these trials

Other methods like virtual or high throughput screening have yielded small molecule HAT inhibitors with diverse structures (Fig. 2). In high throughput screening, large numbers compounds are tested in an enzyme inhibition assays. Originating as hits from a high throughput screenings, isothiazolones have been developed as inhibitors for various subtypes of HATs and proved to inhibit proliferation in cancer cell lines [58–60]. However, most of these isothiazolones are highly reactive towards thiolates, which limits their applicability in biological systems [61]. In virtual screening methods, the crystal structure or homology model of the target protein is used to computationally screen virtual databases of compounds for potential binding. The KAT3B HAT inhibitor 4-acetyl-2-methyl-*N*-morpholino-3,4-dihydro-2H-benzo[b][1, 4]thiazine-7-sulfonamide (Fig. 2, thiazinesulfonamide) was discovered using virtual screening on KAT3B [62]. The inhibitor C646 has been discovered using the same method and is currently the most potent and selective small molecule KAT3B HAT inhibitor [63]. Since its discovery in 2010, this inhibitor has been shown to be active in different cellular models of cancer. Among others, it inhibited proliferation of prostate cancer and melanoma cells, induced cell cycle arrest in leukemia cells, and sensitized lung cancer cells to irradiation [64–67]. Also for other implications, as peritoneal fibrosis, this inhibitor recently showed promising results [68]. This shows that screening methods are valuable for the discovery of HAT inhibitors with novel structures and are expected to yield more inhibitors in the near future.

Another way of inhibiting HAT function, in contrast to inhibiting the acetyltransferase activity, is to target protein–protein interactions between HATs and their interaction partners. HATs interact with many proteins and influence their function, in some cases independent of their acetyltransferase activity. KAT3A, for example, was shown to activate β-catenin, a transcription factor involved in inflammatory signal transduction, independent of its acetyltransferase activity [69]. The inhibitor ICG-001 (Fig. 2) inhibits the interaction between KAT3A and β-catenin, and the inhibition was shown to be selective over the interaction between KAT3B and β-catenin [70]. Studies with this inhibitor revealed a different role for the KAT3A/β-catenin than for the KAT3B/β-catenin interaction in survivin gene transcription [71]. Therefore, protein–protein interaction inhibitors can be used to selectively explore the functions of HATs that are not mediated by the enzymatic acetyltransferase activity. These studies additionally revealed anti-cancer activity in colon carcinoma models for this inhibitor, showing that inhibition of the KAT3A/β-catenin interaction has therapeutic potential.

Several HATs (KAT2A and 2B, KAT3A and 3B) contain a bromodomain, which can bind specifically to acetylated lysine residues. Bromodomain inhibitors target this interaction by preventing the binding of the acetylated lysine to the bromodomain [72]. HAT bromodomain inhibitors have been developed for KAT3A and KAT2B, including the natural product ischemin, a set of cyclic peptides and small molecule N1-aryl-propane-1,3-diamine derivatives [73–75] (Fig. 2). In contrast to the aforementioned inhibitors, these inhibitors do not seem to have potential as anti-cancer agents. The cyclic peptides were developed as inhibitors of the tumor suppressor protein p53, having opposite function as anti-cancer agents [74]. Ischemin inhibited apoptosis in cardiomyocytes, showing potential as therapeutic in myocardial ischemia and [73] the small molecule N1-aryl-propane-1,3-diamine derivatives showed an inhibitory effect on HIV-1 replication, opening possibilities as anti-viral agents [75]. This shows that HAT inhibitors have more potential than anti-cancer agents alone and can possibly be used as therapeutics for many more indications.

A small number of positive modulators or activators of HATs have been described (Fig. 2). The KAT3B selective activator N-(4-chloro-3-trifluoromethyl-phenyl)-2-ethoxy-6-pentadecyl-benzamide (CTPB) was derived from the natural product HAT inhibitor anacardic acid and was shown to activate gene transcription [46]. The activator TTK21 was also based on a salicylic acid structure but was shown to activate both KAT3A and 3B. This activator improved memory duration in mice and was suggested to have opportunities for application in brain disease [76]. Another anacardic acid-based KAT2B activator is the pentadecylidenemalonate SPV106. Interestingly, this compound activates KAT2B but was shown to inhibit KAT3A and 3B [77]. This HAT modulator has been shown to have a positive effect in models of cardiovascular disease, diabetes, wound repair, and the extinction of conditioned fear [78–82]. These examples show that both for inhibitors and activators or mixed activator/inhibitors of HATs, there may be future clinical applications (Table 2).

### Catalytic mechanism—challenges in substrate conversion

HATs catalyze the acetylation of lysine residues using the cofactor Ac-CoA as an acetyl donor. HATs are therefore bi-substrate enzymes, i.e., they bind and convert two substrates in the process of catalysis. Although all HATs acetylate lysine residues and use Ac-CoA as a cofactor, the mechanism of catalysis differs. In theory, there are three standard catalytic mechanisms for bi-substrate enzymes: (i) a random-order ternary complex mechanism, (ii) a compulsory-order ternary complex mechanism, or (iii) a ping-pong mechanism. In a random-order ternary complex mechanism, either substrate can bind first to the enzyme, in a random order. The acetyl group is directly transferred from Ac-CoA to the lysine residue upon formation of the ternary complex by binding of the second substrate. In a compulsory-order ternary complex mechanism, a ternary complex is formed, but one of the substrates has to bind first before the other substrates can bind. In both mechanisms, catalysis depends on the presence of a general base, such as glutamic acid, which facilitates the nucleophilic attack on the Ac-CoA thioester by deprotonating the lysine residue. In a ping-pong mechanism, Ac-CoA binds first and the acetyl group is transferred to an amino acid in the catalytic site of the enzyme. CoA leaves the enzyme and subsequently the substrate binds, to which the acetyl group is transferred. This mechanism requires, next to a general base, an amino acid in the catalytic site of the enzyme suitable for accepting the acetyl group, which is commonly a cysteine [83]. Knowledge on the catalytic mechanism plays an important role in the characterization and development of small molecule enzyme inhibitors. This has for example been shown for the well-known NAD^+^ dependent liver alcohol dehydrogenase, which operates via a compulsory-order ternary complex mechanism, where NAD^+^ must bind first [84, 85]. The development of inhibitors was greatly aided by knowledge on the catalytic mechanism of the alcohol dehyrogenase. Also, in the case of HATs, definition of the catalytic mechanisms is highly important.

There is evidence that the GNAT family HATs catalyze lysine acetylation by a ternary complex mechanism. These enzymes contain a conserved glutamic acid (KAT2A: Glu-173; KAT2B: Glu-570) in the active site, which can serve as general base that deprotonates the positively charged lysine to allow nucleophilic attack on the Ac-CoA thioester [86]. This mechanism is supported by a kinetic study on KAT2B using two-substrate kinetic analysis and a dead-end inhibitor that mimics CoA, but cannot be converted by the enzyme. The study showed that this enzyme follows a compulsory-order ternary complex mechanism in which Ac-CoA binds first to the enzyme followed by the histone substrate [87]. Therefore, the current consensus is that the GNAT family HATs catalyze lysine acetylation through a compulsory-order ternary complex mechanism.

For MYST family proteins, studies have described different catalytic mechanisms. For the MYST family HAT KAT8, a kinetic study on the recombinant catalytic domain, showed a pattern consistent with a ping-pong mechanism in which the acetyl moiety is transferred onto a residue in the active site of the enzyme. The subsequent binding of Ac-CoA and the histone peptide was confirmed by calorimetric binding measurements [88]. KAT8 contains the conserved glutamic acid, Glu-177, which can act as a general base as well as a cysteine in the catalytic site, Cys143, which is capable of accepting the acetyl moiety in case of a ping-pong mechanism [PDB: 3TOA [89]]. In a study with the catalytic domain of ESA1, a MYST family HAT from yeast that shows close homology to human KAT5 and KAT8, it was shown that cysteine 304 (Cys-304) and glutamic acid 338 (Glu-338) are both essential for enzyme activity. Glu-338 was shown to function as a general base, as in GNAT family HATs [90]. A crystal structure of truncated ESA1 co-crystallized with Ac-CoA showed that the acetyl moiety of Ac-CoA had transferred from the cofactor to Cys-304, supporting a ping-pong mechanism [91]. However, this was countered by a study showing that mutation of Cys-304, in contrast to the aforementioned study, did not impair the activity of the enzyme and kinetic studies showed a pattern indicating catalysis via a ternary complex mechanism [92]. In this study, not the catalytic HAT domain, but full-length ESA1 was used and it was combined with two other proteins forming the piccolo NuA4 complex, which is naturally occurring in yeast. This shows that the catalytic mechanism of ESA1 was influenced by the interaction with other proteins. Therefore, just as the substrate specificity and acetyltransferase activity of HATs is influenced by the incorporation into HAT protein complexes, these complexes may influence the catalytic mechanism as well.

As for MYST family enzymes, the catalytic mechanism for the p300/CBP family depends on the experimental methods applied in the respective study. Based on kinetic measurements with the recombinant full-length enzyme, it was proposed that KAT3B uses a ping-pong mechanism [93]. Studies using an Ac-CoA-based probe that targets cysteine residues showed that the probe bound a cysteine residue in the catalytic domain of KAT3B, which was important for Ac-CoA binding. However, the catalytic activity of KAT3B was not abolished by mutation of this cysteine residue, which would be expected in a ping-pong mechanism [94]. The possibility of a ternary complex mechanism was investigated by comparing the affinity pattern of different bi-substrate inhibitors [95]. In a ternary complex mechanism, inhibitors with a longer peptide part should have better affinity, but in case of KAT3B, it was shown that the shortest inhibitor was most potent. Therefore, it was proposed that KAT3B uses a Theorell–Chance (“hit-and-run”) catalytic mechanism. In the Theorell–Chance mechanism, there is no stable ternary complex. Ac-CoA binds first and subsequently, the peptide substrate binds weakly to the enzyme, allowing the lysine to react with the acetyl group. However, kinetically only the interaction with Ac-CoA is important [95]. In studies on the catalytic mechanism of KAT3B, kinetic measurements, affinity labeling-based probes, substrate mimic inhibitors, crystallization, and mutagenesis studies, resulted in proposals for different mechanisms. This shows that using a single method may not be sufficient to conclude on the catalytic mechanism of HATs.

So far, different studies indicate different catalytic mechanisms for specific HATs. The use of different constructs of the HAT enzymes and the use of different methods leads to different proposed catalytic mechanisms. Table 3 summarizes the proposed catalytic mechanisms for different HAT families, the enzyme constructs that are used, and methods that are applied. We note that, independent from the mechanism found, all HATs seem to conserve both a glutamic acid, which can function as a general base to deprotonate the lysine residue, and a cysteine residue, which can serve as acetyl acceptor in the formation of acetylated enzyme intermediate in a ping-pong mechanism. Nevertheless, despite the presence of this cysteine residue, it is not in all cases critical for catalysis. Apparently, the methods used in these studies cannot distinguish between the types of mechanisms for these HATs, which may indicate that both mechanisms could occur, depending on the methods used and the conditions applied. If the energetic profile for the different catalytic mechanisms is very similar, small changes in assay conditions could lead to the observation of different catalytic mechanisms. This may indicate that HATs are flexible enzymes which can act via different catalytic mechanisms under different conditions.

**Table 3:** Reported catalytic mechanisms for the different HAT families, the enzyme constructs used, and experimental methods applied

Family	Mechanism	Enzyme (amino acids)	Methods	Reference
GNAT	Compulsory-order ternary complex mechanism	KAT2A HAT domain (99–262)	Mutagenesis studies, biochemical studies	[86]
		KAT2B catalytic domain (493–676) and full-length	Kinetic analysis, dead-end substrate mimic inhibitor	[45]
MYST	Ping-pong mechanism	Yeast ESA1 HAT domain (160–435)	Crystal structure, mutagenesis	[91]
		KAT8 C-terminal (125–458)	Kinetic analysis, calorimetric binding studies	[88]
	Ternary complex mechanism	Yeast ESA1 full-length and picNuA4 complex	Kinetic analysis, mutagenesis studies	[92]
p300/CBP	Ping-pong mechanism	KAT3B full-length	Kinetic analysis	[93]
	Theorell-Chance mechanism	KAT3B catalytic domain (1284–1673)	Chemical probe	[94]
		KAT3B semi-synthetic heterodimeric HAT domain (1287–1652)	Crystal structure, bi-substrate inhibitor, mutagenesis, kinetic analysis	[95]

### HAT inhibitors—challenges in inhibitor kinetics

The fact that HATs are bi-substrate enzymes does not only affect the analysis of their catalytic mechanisms but also has consequences for the development of small molecule inhibitors for these enzymes. To characterize the potency of such inhibitors, they are often tested in steady-state enzyme inhibition assays. From these assays, the concentrations that give 50 % inhibition of the enzyme activity (IC_50_) are derived. However, these values depend on the assay conditions, and therefore, reporting the inhibitory potency (*K*_i_) is preferred. The *K*_i_ value allows for better reproducibility between enzyme inhibition assays and is therefore important for further development of potent and selective inhibitors. In case of a single-substrate enzyme and a competitive inhibitor, the IC_50_ can be corrected for the assay conditions using the Cheng–Prusoff equation using the substrate concentration and the Michaelis constant (*K*_m_) of the substrate [96]. However, in case of bi-substrate enzymes like HATs, additional factors influence the IC_50_, namely the catalytic mechanism, the concentration of both substrates, and their respective Michaelis constants [83, 96]. The KAT3B inhibitor C646 was shown to be competitive with Ac-CoA and non-competitive with the histone substrate [63]. Further studies showed that the level of inhibition by C646 was not time-dependent and that pre-incubation did not influence the level of inhibition, showing that it is a reversible inhibitor. A *K*_i_ value was derived from the Dixon plots, which seems to be justified considering the described mechanism. There are, however, few reports on the calculation of *K*_i_ values in case of a Theorell–Chance mechanism, except in case of bi-substrate analogue dead-end inhibitors [97]. The *K*_i_ value of C646 was shown to be 3.2-fold lower than the IC_50_, showing the significance of the calculation of this value. Although not aimed at calculating the inhibitory potency, an interesting mechanistic investigation of garcinol and two derivatives used calorimetric binding studies and kinetic evaluations to propose a mechanism for the binding of these inhibitors [98]. An enzyme kinetic study on inhibition of the MYST family HAT KAT8 by the natural product HAT inhibitor anacardic acid revealed a more complicated binding model [88]. This enzyme proved to catalyze histone acetylation via a ping-pong mechanism, and according to the enzyme kinetics, the inhibitor proved to bind to the acetylated enzyme intermediate. This information enabled the calculation of the *K*_i_ value for KAT8 inhibition by anacardic acid and several derivatives, using an equation reported by Cheng and Prusoff [96]. Also in this case, the *K*_i_ values of anacardic acid were more than threefold lower compared to the IC_50_ values under the applied assay conditions. These examples underline the importance of the determination of the kinetic mechanisms and the calculation of *K*_i_ values.

**Fig. 3:**
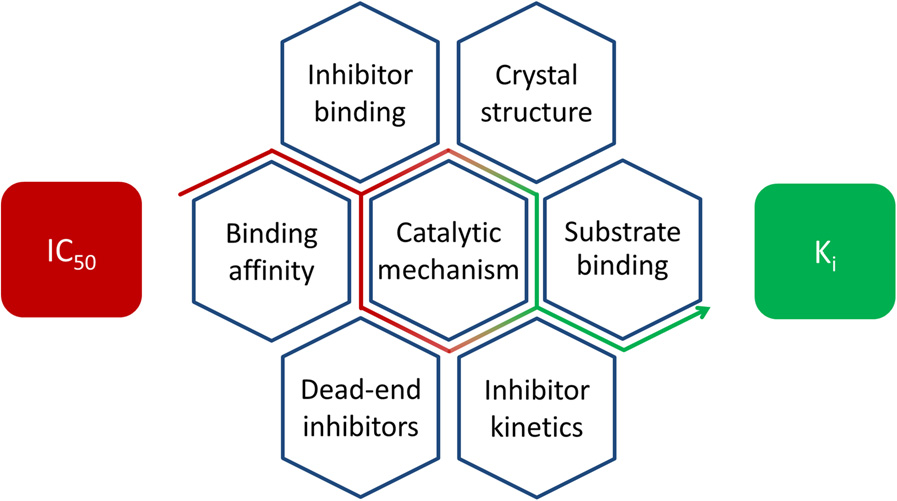
Challenges to get from the concentration of inhibitor that gives 50 % of inhibition (IC_50_) to the assay independent inhibitory potency (*K*_i_) for a HAT inhibitor. In case of bi-substrate enzymes like HATs, many factors need to be considered when calculating the inhibitory potency from the IC_50_. Kinetic studies combined with affinity studies, crystal structures, dead-end inhibitors, and studies on the catalytic mechanism of HATs aid in deriving a *K*_i_ for HAT inhibitors

Considering the dependence of the IC_50_ values on the *K*_m_ values and concentrations of both substrates, it is clear that IC_50_ values are prone to variations between different studies and assay set-ups. Nevertheless, very few studies have currently been reported in which the mechanism of inhibition and *K*_i_ values of existing HAT inhibitors have been calculated. This does, however, pose problems for further development of HAT inhibitors. It is, for example, not possible to compare the potencies of the new inhibitors with the potencies of existing inhibitors, unless exactly the same assays with the same conditions are used. In addition, it is often overlooked that it is not possible to conclude on selectivity of an inhibitor based on IC_50_ values, especially in the case of bi-substrate enzymes in which IC_50_ values strongly depend on both substrates and the catalytic mechanism. Therefore, it is important to investigate the enzyme kinetics of HAT inhibitors carefully, using multiple methods (Fig. 3). This will aid in deriving a *K*_i_ value for the inhibitors and increase the understanding of HAT enzymes, which will facilitate the further development of novel potent and specific HAT inhibitors.

## Conclusions

HATs are upcoming targets in drug discovery with potential applications in many disease models. Nevertheless, as our knowledge is progressing, the challenges in targeting these enzymes become more and more clear. One of the challenges is that HATs have various cellular substrates ranging from histones and transcription factors to enzymes and nuclear receptors. In addition, they operate as part of multi-protein complexes, which determine their functions, their enzymatic activities and their substrate specificities. This complicates the translation of studies on recombinant enzymes to cellular studies and more advanced (in vivo) disease models. The challenges in the development of small molecule inhibitors of HAT activity have been addressed over recent years, but most of the resulting inhibitors still suffer from undesired properties such as anti-oxidant activity, instability in a cellular environment, low potency, or lack of selectivity between HAT subtypes and other enzymes.

Another important challenge is the understanding of the catalytic mechanisms of HAT activity in relation to enzyme kinetics of small molecule HAT inhibitors. As bi-substrate enzymes, HATs catalyze the reaction between two substrates, the cofactor Ac-CoA and the lysine residue on the target protein. The catalytic mechanism, by which these substrates are converted by HATs, is influenced by the enzyme length and the experimental methods applied to measure the enzyme activity. Furthermore, the presence of other proteins that can modulate HAT activity by the formation of protein–protein complexes can also affect the catalytic mechanism. Despite the difficulties of investigating the catalytic mechanism, knowledge on the catalytic mechanism is very important for further understanding of how HATs work and for the development of inhibitors that are potent and selective. Combined with the catalytic mechanism of the HAT enzyme, inhibitor kinetics can enable the calculation of assay independent inhibitory constants (*K*_i_). The ability to calculate the inhibitory potency of inhibitors will enable comparison with existing HAT inhibitors and determination of the selectivity. This will greatly enhance the discovery of HAT inhibitors and improve their chances to be taken into further development as research tools or therapeutic agents.

## Abbreviations

Ac-CoA: acetyl coenzyme A
CBP: CREB-binding protein
c-MYC: Myc proto-oncogene protein
CREB: cAMP response element binding protein
Gcn5: general control of amino acid synthesis protein 5
GNAT: Gcn5-related *N*-acetyltransferases
H4K16: lysine 16 on histone H4
HAT: histone acetyltransferase
HBO: histone acetyltransferase binding to ORC1
HDAC: histone deacetylase
IC_50_: concentration of inhibitor that gives 50 % of inhibition
KAT: lysine (K) acetyltransferase
*K*_i_: inhibitory potency
*K*_m_: Michaelis constant—concentration of substrate that give 50 % of maximum velocity
Lys-CoA: lysine-CoA, bi-substrate inhibitor
MORF: MOZ-related factor
MOZ: monocytic leukemic zinc finger
MYST: MOZ, YBF2/SAS3, SAS2, and TIP60
NF-κB: nuclear factor kappa-light-chain-enhancer of activated B cells
PCAF: p300/CBP-associated factor
TIP60: 60 kDa Tat-interactive protein

## References

[1] Iyer A, Fairlie DP, Brown L (2012). Lysine acetylation in obesity, diabetes and metabolic disease. Immunol Cell Biol.

[2] Khan SN, Khan AU (2010). Role of histone acetylation in cell physiology and diseases: an update. Clin Chim Acta.

[3] Choudhary C, Kumar C, Gnad F, Nielsen ML, Rehman M, Walther TC (2009). Lysine acetylation targets protein complexes and co-regulates major cellular functions. Science.

[4] Grunstein M (1997). Histone acetylation in chromatin structure and transcription. Nature.

[5] Strahl BD, Allis CD (2000). The language of covalent histone modifications. Nature.

[6] Patel JH, Du Y, Ard PG, Phillips C, Carella B, Chen CJ (2004). The c-MYC oncoprotein is a substrate of the acetyltransferases hGCN5/PCAF and TIP60. Mol Cell Biol.

[7] Grossman SR (2001). p300/CBP/p53 interaction and regulation of the p53 response. Eur J Biochem.

[8] Ghizzoni M, Haisma HJ, Maarsingh H, Dekker FJ (2011). Histone acetyltransferases are crucial regulators in NF-kappaB mediated inflammation. Drug Discov Today.

[9] Han Y, Jin YH, Kim YJ, Kang BY, Choi HJ, Kim DW (2008). Acetylation of Sirt2 by p300 attenuates its deacetylase activity. Biochem Biophys Res Commun.

[10] Sharma M, Zarnegar M, Li X, Lim B, Sun Z (2000). Androgen receptor interacts with a novel MYST protein, HBO1. J Biol Chem.

[11] Yang X (2004). The diverse superfamily of lysine acetyltransferases and their roles in leukemia and other diseases. Nucleic Acids Res.

[12] Current perspectives on role of chromatin modifications and deacetylases in lung inflammation in COPD. COPD. 2009;6(4):291-7. 10.1080/15412550903049132 PMC2760053 19811389

[13] Sheikh BN (2014). Crafting the brain—role of histone acetyltransferases in neural development and disease. Cell Tissue Res.

[14] Bianco-Miotto T, Chiam K, Buchanan G, Jindal S, Day TK, Thomas M (2010). Global levels of specific histone modifications and an epigenetic gene signature predict prostate cancer progression and development. Cancer Epidemiol Biomarkers Prev.

[15] Bai X, Wu L, Liang T, Liu Z, Li J, Li D (2008). Overexpression of myocyte enhancer factor 2 and histone hyperacetylation in hepatocellular carcinoma. J Cancer Res Clin Oncol.

[16] Liu B, Cheng J, Zhang X, Wang R, Zhang W, Lin H (2010). Global histone modification patterns as prognostic markers to classify glioma patients. Cancer Epidemiol Biomarkers Prev.

[17] Gayther SA, Batley SJ, Linger L, Bannister A, Thorpe K, Chin SF (2000). Mutations truncating the EP300 acetylase in human cancers. Nat Genet.

[18] Ishihama K, Yamakawa M, Semba S, Takeda H, Kawata S, Kimura S (2007). Expression of HDAC1 and CBP/p300 in human colorectal carcinomas. J Clin Pathol.

[19] Liu L, Scolnick DM, Trievel RC, Zhang HB, Marmorstein R, Halazonetis TD (1999). p53 sites acetylated in vitro by PCAF and p300 are acetylated in vivo in response to DNA damage. Mol Cell Biol.

[20] Liu C, Lu J, Tan J, Li L, Huang B (2004). Human interleukin-5 expression is synergistically regulated by histone acetyltransferase CBP/p300 and transcription factors C/EBP, NF-AT and AP-1. Cytokine.

[21] Schmeck B, Lorenz J, N’Guessan PD, Opitz B, van Laak V, Zahlten J (2008). Histone acetylation and flagellin are essential for Legionella pneumophila-induced cytokine expression. J Immunol.

[22] Gingras S, Simard J, Groner B, Pfitzner E (1999). p300/CBP is required for transcriptional induction by interleukin-4 and interacts with Stat6. Nucleic Acids Res.

[23] Sheppard KA, Rose DW, Haque ZK, Kurokawa R, McInerney E, Westin S (1999). Transcriptional activation by NF-kappaB requires multiple coactivators. Mol Cell Biol.

[24] Ashburner BP, Westerheide SD, Baldwin AS (2001). The p65 (RelA) subunit of NF-kappaB interacts with the histone deacetylase (HDAC) corepressors HDAC1 and HDAC2 to negatively regulate gene expression. Mol Cell Biol.

[25] Miao F, Gonzalo IG, Lanting L, Natarajan R (2004). In vivo chromatin remodeling events leading to inflammatory gene transcription under diabetic conditions. J Biol Chem.

[26] Gunawardhana LP, Gibson PG, Simpson JL, Powell H, Baines KJ (2014). Activity and expression of histone acetylases and deacetylases in inflammatory phenotypes of asthma. Clin Exp Allergy.

[27] Henderson WR, Chi EY, Ye X, Nguyen C, Tien Y, Zhou B (2010). Inhibition of Wnt/ß-catenin/CREB binding protein (CBP) signaling reverses pulmonary fibrosis. Proc Natl Acad Sci.

[28] Bastiaansen AJ, Ewing MM, de Boer HC, van der Pouw Kraan TC, de Vries MR, Peters EA (2013). Lysine acetyltransferase PCAF is a key regulator of arteriogenesis. Arterioscler Thromb Vasc Biol.

[29] Roelfsema JH, Peters DJ (2007). Rubinstein-Taybi syndrome: clinical and molecular overview. Expert Rev Mol Med.

[30] Gupta A, Guerin-Peyrou TG, Sharma GG, Park C, Agarwal M, Ganju RK (2008). The mammalian ortholog of Drosophila MOF that acetylates histone H4 lysine 16 is essential for embryogenesis and oncogenesis. Mol Cell Biol.

[31] Tanaka Y, Naruse I, Hongo T, Xu M, Nakahata T, Maekawa T (2000). Extensive brain hemorrhage and embryonic lethality in a mouse null mutant of CREB-binding protein. Mech Dev.

[32] Yao TP, Oh SP, Fuchs M, Zhou ND, Ch’ng LE, Newsome D (1998). Gene dosage-dependent embryonic development and proliferation defects in mice lacking the transcriptional integrator p300. Cell.

[33] Furdas SD, Kannan S, Sippl W, Jung M (2012). Small molecule inhibitors of histone acetyltransferases as epigenetic tools and drug candidates. Arch Pharm (Weinheim).

[34] Dekker FJ, Haisma HJ (2009). Histone acetyl transferases as emerging drug targets. Drug Discov Today.

[35] Parthun MR, Widom J, Gottschling DE (1996). The major cytoplasmic histone acetyltransferase in yeast: links to chromatin replication and histone metabolism. Cell.

[36] Ogryzko VV, Schiltz RL, Russanova V, Howard BH, Nakatani Y (1996). The transcriptional coactivators p300 and CBP are histone acetyltransferases. Cell.

[37] Wang YL, Faiola F, Xu M, Pan S, Martinez E (2008). Human ATAC is a GCN5/PCAF-containing acetylase complex with a novel NC2-like histone fold module that interacts with the TATA-binding protein. J Biol Chem.

[38] Smith ER, Pannuti A, Gu W, Steurnagel A, Cook RG, Allis CD (2000). The Drosophila MSL complex acetylates histone H4 at lysine 16, a chromatin modification linked to dosage compensation. Mol Cell Biol.

[39] Lee KK, Workman JL (2007). Histone acetyltransferase complexes: one size doesn’t fit all. Nat Rev Mol Cell Biol.

[40] Li X, Wu L, Corsa CA, Kunkel S, Dou Y (2009). Two mammalian MOF complexes regulate transcription activation by distinct mechanisms. Mol Cell.

[41] Smith ER, Cayrou C, Huang R, Lane WS, Cote J, Lucchesi JC (2005). A human protein complex homologous to the Drosophila MSL complex is responsible for the majority of histone H4 acetylation at lysine 16. Mol Cell Biol.

[42] Grant PA, Eberharter A, John S, Cook RG, Turner BM, Workman JL (1999). Expanded lysine acetylation specificity of Gcn5 in native complexes. J Biol Chem.

[43] Lau OD, Kundu TK, Soccio RE, Ait-Si-Ali S, Khalil EM, Vassilev A (2000). HATs off: selective synthetic inhibitors of the histone acetyltransferases p300 and PCAF. Mol Cell.

[44] Yang C, Ngo L, Zheng YG (2014). Rational design of substrate-based multivalent inhibitors of the histone acetyltransferase Tip60. ChemMedChem.

[45] Lau OD, Courtney AD, Vassilev A, Marzilli LA, Cotter RJ, Nakatani Y (2000). p300/CBP-associated factor histone acetyltransferase processing of a peptide substrate. Kinetic analysis of the catalytic mechanism. J Biol Chem.

[46] Balasubramanyam K, Swaminathan V, Ranganathan A, Kundu TK (2003). Small molecule modulators of histone acetyltransferase p300. J Biol Chem.

[47] Balasubramanyam K, Varier RA, Altaf M, Swaminathan V, Siddappa NB, Ranga U (2004). Curcumin, a novel p300/CREB-binding protein-specific inhibitor of acetyltransferase, represses the acetylation of histone/nonhistone proteins and histone acetyltransferase-dependent chromatin transcription. J Biol Chem.

[48] Balasubramanyam K, Altaf M, Varier RA, Swaminathan V, Ravindran A, Sadhale PP (2004). Polyisoprenylated benzophenone, garcinol, a natural histone acetyltransferase inhibitor, represses chromatin transcription and alters global gene expression. J Biol Chem.

[49] Masuoka N, Kubo I (2004). Characterization of xanthine oxidase inhibition by anacardic acids. Biochim Biophys Acta.

[50] Wang D, Girard TJ, Kasten TP, LaChance RM, Miller-Wideman M, Durley RC (1998). Inhibitory activity of unsaturated fatty acids and anacardic acids toward soluble tissue factor-factor VIIa complex. J Nat Prod.

[51] Sun Y, Jiang X, Chen S, Price BD (2006). Inhibition of histone acetyltransferase activity by anacardic acid sensitizes tumor cells to ionizing radiation. FEBS Lett.

[52] Oike T, Ogiwara H, Torikai K, Nakano T, Yokota J, Kohno T (2012). Garcinol, a histone acetyltransferase inhibitor, radiosensitizes cancer cells by inhibiting non-homologous end joining. Int J Radiat Oncol Biol Phys.

[53] Tsai M, Chiou Y, Chiou L, Ho C, Pan M (2014). Garcinol suppresses inflammation-associated colon carcinogenesis in mice. Mol Nutr Food Res.

[54] Ye X, Yuan L, Zhang L, Zhao J, Zhang CM, Deng HY (2014). Garcinol, an acetyltransferase inhibitor, suppresses proliferation of breast cancer cell line MCF-7 promoted by 17beta-estradiol. Asian Pac J Cancer Prev.

[55] Priyadarsini KI (2014). The chemistry of curcumin: from extraction to therapeutic agent. Molecules.

[56] Gao C, Bourke E, Scobie M, Famme MA, Koolmeister T, Helleday T (2014). Rational design and validation of a Tip60 histone acetyltransferase inhibitor. Sci Rep.

[57] Milite C, Feoli A, Sasaki K, La Pietra V, Balzano AL, Marinelli L (2015). A novel cell-permeable, selective, and noncompetitive inhibitor of KAT3 histone acetyltransferases from a combined molecular pruning/classical isosterism approach. J Med Chem.

[58] Stimson L, Rowlands MG, Newbatt YM, Smith NF, Raynaud FI, Rogers P (2005). Isothiazolones as inhibitors of PCAF and p300 histone acetyltransferase activity. Mol Cancer Ther.

[59] Gorsuch S, Bavetsias V, Rowlands MG, Aherne GW, Workman P, Jarman M (2009). Synthesis of isothiazol-3-one derivatives as inhibitors of histone acetyltransferases (HATs). Bioorg Med Chem.

[60] Dekker FJ, Ghizzoni M, Van der Meer N, Wisastra R, Haisma HJ (2009). Inhibition of the PCAF histone acetyl transferase and cell proliferation by isothiazolones. Bioorg Med Chem.

[61] Wisastra R, Ghizzoni M, Maarsingh H, Minnaard AJ, Haisma HJ, Dekker FJ (2011). Isothiazolones; thiol-reactive inhibitors of cysteine protease cathepsin B and histone acetyltransferase PCAF. Org Biomol Chem.

[62] Zeng FQ, Peng SM, Li L, Mu L, Zhang Z (2013). Structure-based identification of drug-like inhibitors of p300 histone acetyltransferase. Acta Pharmaceutica Sinica.

[63] Bowers EM, Yan G, Mukherjee C, Orry A, Wang L, Holbert MA (2010). Virtual ligand screening of the p300/CBP histone acetyltransferase: identification of a selective small molecule inhibitor. Chem Biol.

[64] Yan G, Eller MS, Elm C, Larocca CA, Ryu B, Panova IP (2013). Selective inhibition of p300 HAT blocks cell cycle progression, induces cellular senescence, and inhibits the DNA damage response in melanoma cells. J Invest Dermatol.

[65] Santer FR, Hoschele PP, Oh SJ, Erb HH, Bouchal J, Cavarretta IT (2011). Inhibition of the acetyltransferases p300 and CBP reveals a targetable function for p300 in the survival and invasion pathways of prostate cancer cell lines. Mol Cancer Ther.

[66] Gao XN, Lin J, Ning QY, Gao L, Yao YS, Zhou JH (2013). A histone acetyltransferase p300 inhibitor C646 induces cell cycle arrest and apoptosis selectively in AML1-ETO-positive AML cells. PLoS One.

[67] Oike T, Komachi M, Ogiwara H, Amornwichet N, Saitoh Y, Torikai K (2014). C646, a selective small molecule inhibitor of histone acetyltransferase p300, radiosensitizes lung cancer cells by enhancing mitotic catastrophe. Radiother Oncol.

[68] Yang Y, Liu K, Liang Y, Chen Y, Chen Y, Gong Y (2015). Histone acetyltransferase inhibitor C646 reverses epithelial to mesenchymal transition of human peritoneal mesothelial cells via blocking TGF-beta1/Smad3 signaling pathway in vitro. Int J Clin Exp Pathol.

[69] Hecht A, Vleminckx K, Stemmler MP, van Roy F, Kemler R (2000). The p300/CBP acetyltransferases function as transcriptional coactivators of beta-catenin in vertebrates. EMBO.

[70] Emami KH, Nguyen C, Ma H, Kim DH, Jeong KW, Eguchi M (2004). A small molecule inhibitor of beta-catenin/CREB-binding protein transcription [corrected. Proc Natl Acad Sci U S A.

[71] Ma H, Nguyen C, Lee KS, Kahn M (2005). Differential roles for the coactivators CBP and p300 on TCF/beta-catenin-mediated survivin gene expression. Oncogene.

[72] Muller S, Filippakopoulos P, Knapp S (2011). Bromodomains as therapeutic targets. Expert Rev Mol Med.

[73] Borah JC, Mujtaba S, Karakikes I, Zeng L, Muller M, Patel J (2011). A small molecule binding to the coactivator CREB-binding protein blocks apoptosis in cardiomyocytes. Chem Biol.

[74] Gerona-Navarro G, Rodríguez-Fernández Y, Mujtaba S, Frasca A, Patel J, Zeng L (2011). Rational design of cyclic peptide modulators of the transcriptional coactivator CBP: a new class of p53 inhibitors. J Am Chem Soc.

[75] Wang Q, Wang R, Zhang B, Zhang S, Zheng Y, Wang Z (2013). Small organic molecules targeting PCAF bromodomain as potent inhibitors of HIV-1 replication. Med Chem Commun.

[76] Chatterjee S, Mizar P, Cassel R, Neidl R, Selvi BR, Mohankrishna DV (2013). A novel activator of CBP/p300 acetyltransferases promotes neurogenesis and extends memory duration in adult mice. J Neurosci.

[77] Sbardella G, Castellano S, Vicidomini C, Rotili D, Nebbioso A, Miceli M (2008). Identification of long chain alkylidenemalonates as novel small molecule modulators of histone acetyltransferases. Bioorg Med Chem Lett.

[78] Colussi C, Scopece A, Vitale S, Spallotta F, Mattiussi S, Rosati J (2012). P300/CBP associated factor regulates nitroglycerin-dependent arterial relaxation by N(epsilon)-lysine acetylation of contractile proteins. Arterioscler Thromb Vasc Biol.

[79] Vecellio M, Spallotta F, Nanni S, Colussi C, Cencioni C, Derlet A (2014). The histone acetylase activator pentadecylidenemalonate 1b rescues proliferation and differentiation in the human cardiac mesenchymal cells of type 2 diabetic patients. Diabetes.

[80] Colussi C, Rosati J, Straino S, Spallotta F, Berni R, Stilli D (2011). Nepsilon-lysine acetylation determines dissociation from GAP junctions and lateralization of connexin 43 in normal and dystrophic heart. Proc Natl Acad Sci U S A.

[81] Spallotta F, Cencioni C, Straino S, Sbardella G, Castellano S, Capogrossi MC (2013). Enhancement of lysine acetylation accelerates wound repair. Commun Integr Biol.

[82] Wei W, Coelho CM, Li X, Marek R, Yan S, Anderson S (2012). p300/CBP-associated factor selectively regulates the extinction of conditioned fear. J Neurosci.

[83] Copeland RA. Enzyme Reactions with Multiple Substrates. Enzymes: a practical introduction to structure, mechanism, and data analysis. 2nd ed. New York: Wiley; 2004. p. 350-66.

[84] Wratten CC, Cleland WW (1963). Product inhibition studies on yeast and liver alcohol dehydrogenases*. Biochemistry (N Y).

[85] Koppaka V, Thompson DC, Chen Y, Ellermann M, Nicolaou KC, Juvonen RO (2012). Aldehyde dehydrogenase inhibitors: a comprehensive review of the pharmacology, mechanism of action, substrate specificity, and clinical application. Pharmacol Rev.

[86] Tanner KG, Trievel RC, Kuo M, Howard RM, Berger SL, Allis CD (1999). Catalytic mechanism and function of invariant glutamic acid 173 from the histone acetyltransferase GCN5 transcriptional coactivator. J Biol Chem.

[87] Tanner KG, Langer MR, Kim Y, Denu JM (2000). Kinetic mechanism of the histone acetyltransferase GCN5 from yeast. J Biol Chem.

[88] Wapenaar H, van der Wouden PE, Groves MR, Rotili D, Mai A, Dekker FJ (2015). Enzyme kinetics and inhibition of histone acetyltransferase KAT8. Eur J Med Chem.

[89] Yuan H, Rossetto D, Mellert H, Dang W, Srinivasan M, Johnson J (2012). MYST protein acetyltransferase activity requires active site lysine autoacetylation. EMBO J.

[90] Yan Y, Barlev NA, Haley RH, Berger SL, Marmorstein R (2000). Crystal structure of yeast Esa1 suggests a unified mechanism for catalysis and substrate binding by histone acetyltransferases. Mol Cell.

[91] Yan Y, Harper S, Speicher DW, Marmorstein R (2002). The catalytic mechanism of the ESA1 histone acetyltransferase involves a self-acetylated intermediate. Nat Struct Biol.

[92] Berndsen CE, Albaugh BN, Tan S, Denu JM (2007). Catalytic mechanism of a MYST family histone acetyltransferase. Biochemistry.

[93] Thompson PR, Kurooka H, Nakatani Y, Cole PA (2001). Transcriptional coactivator protein p300: kinetic characterization of its histone acetyltransferase activity. J Biol Chem.

[94] Hwang Y, Thompson PR, Wang L, Jiang L, Kelleher NL, Cole PA (2007). A selective chemical probe for coenzyme-A-requiring enzymes. Angew Chem Int Ed.

[95] Liu X, Wang L, Zhao K, Thompson PR, Hwang Y, Marmorstein R (2008). The structural basis of protein acetylation by the p300/CBP transcriptional coactivator. Nature.

[96] Cheng Y, Prusoff WH (1973). Relationship between the inhibition constant (*K*_I_) and the concentration of inhibitor which causes 50 per cent inhibition (*I*_50_) of an enzymatic reaction. Biochem Pharmacol.

[97] Yu M, Magalhaes ML, Cook PF, Blanchard JS (2006). Bisubstrate inhibition: theory and application to N-acetyltransferases. Biochemistry.

[98] Arif M, Pradhan SK, Thanuja GR, Vedamurthy BM, Agrawal S, Dasgupta D (2009). Mechanism of p300 specific histone acetyltransferase inhibition by small molecules. J Med Chem.

